# Supplementary material for: An unexpected role of neutrophils in clearing apoptotic hepatocytes in vivo
Source: eLife. 2023 Sep 20;12:RP86591. doi: 10.7554/eLife.86591 (PMC10511239; doi:10.7554/eLife.86591)
Supplement: Figure 4—figure supplement 1—source data 1. [file elife-86591-fig4-figsupp1-data1.pdf]

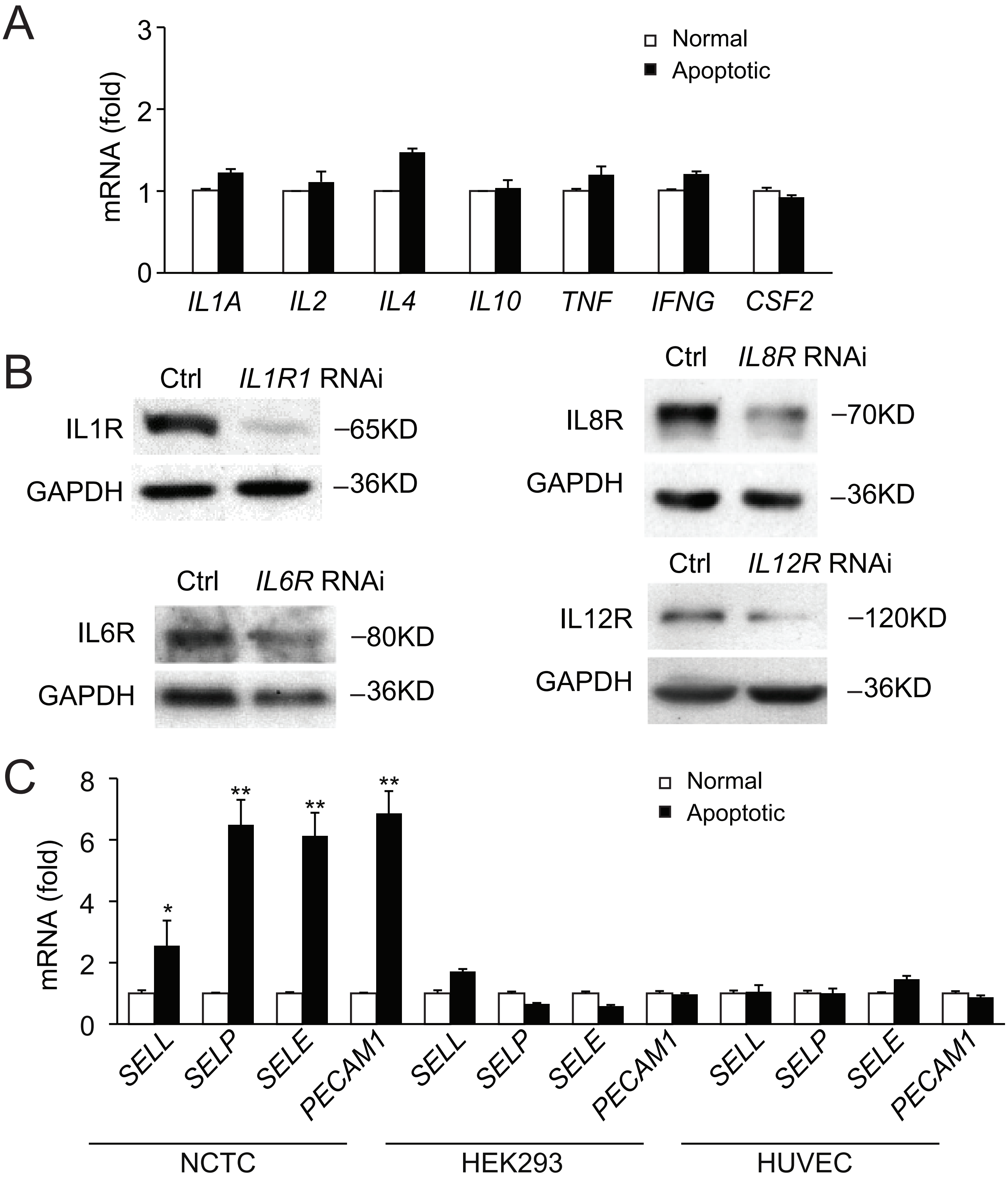

Cytokines and cell surface receptors for perferocytosis. (A) Cytokines secreted by normal nonapoptotic and apoptotic NCTC cells are not significantly changed, includig IL-1α, IL-2, IL-4, IL-10, TNF-α, IFN-γ, and GM-CSF. (B) Immunblots of target proteins (IL-1β, IL-8, IL-6, IL-12 receptors) in non-treated (Ctrl) and RNAi-treated HL60 cells. RNAi knockdown efficiency is ranged from 70-90%. (b) Cell surface receptors in apoptotic and nonapoptotic NCTC cells, HEK293 cells or HUVECs. Data are from (A, C) or representative of (B) three independent experiments (mean and s.e.m. in A, C).
